# Right and left amygdalae activation in patients with major depression receiving antidepressant treatment, as revealed by fMRI

**DOI:** 10.1186/1744-9081-10-36

**Published:** 2014-10-08

**Authors:** Yen-Ting Chen, Min-Wei Huang, I-Chung Hung, Hsien-Yuan Lane, Chun-Ju Hou

**Affiliations:** Department of Electrical Engineering, Southern Taiwan University of Science and Technology, Tainan, 71005 Taiwan; Bali Psychiatric Center, Ministry of Health and Welfare, New Taipei, 24936 Taiwan; Department of Psychiatry, Chiayi Branch, Taichung Veterans General Hospital, Chia-Yi, 60090 Taiwan; Graduate Institute of Clinical Medical Science, China Medical University, Taichung, 40402 Taiwan

**Keywords:** Amygdala, DBOLD_LR_ signal, Emotion, Major Depression, Antidepressants

## Abstract

**Background:**

A differential contribution of the right and left amygdalae to affective information processing has been proposed. However, the direction of this lateralization has not been confirmed. In this study, we used a pre- and post-treatment (escitalopram) design to analyze the relative differences between neural activity in the right and left amygdalae during exposure to emotional stimuli in currently depressed patients. To the best of our knowledge, this study is to compare neural activity between the left and right amygdalae in people with depression. Our findings could lead to the development of parameters or biomarkers for depressive symptoms and treatment response.

**Methods:**

We used a pre–post-test design without a control group. Twenty currently depressed participants underwent an emotion processing task during fMRI. These participants were then treated with an antidepressant for 6 weeks. We used amygdala region-of-interest analysis to evaluate the hemodynamic response during exposure to colored emotional pictures.

**Results:**

In total, thirteen of the 20 participants were placed into a separate group based on degree of response to antidepressants. The partial response group had an averaged HDRS score of 10.75 ± 2.25 and an averaged DBOLD_LR_ signal of 189.18 ± 140.23 (*m*_1_ = 8), and the remitted group had an averaged HDRS score of 4.80 ± 1.64 and an averaged DBOLD_LR_ signal of 421.26 ± 109.19 (*m*_2_ = 5). Each individual had lateralized amygdala activity, and the direction of asymmetry persisted following treatment. Amygdala responses to four types of emotional stimuli did not significantly change (p > 0.05) with treatment in either the right or the left amygdala. However, the difference in neural activity between the right and left amygdalae was greater after treatment, and the variation in neural activity was larger in the left amygdala.

**Conclusions:**

We found that the response between the right and left amygdala did not differ in terms of time series, although activity increased after pharmaceutical treatment for each emotion tested. In the future, changes in BOLD signals as revealed by fMRI might be useful in evaluating the clinical manifestation of major depression.

## Background

Both animal studies and human studies with non-invasive techniques have indicated that the amygdala plays a crucial role in emotional processing [1–3]. Memories about a stimulus that are stored in the hippocampus can predict activation in the amygdala in response to the stimulus, indicating that the amygdala receives sensory input [4]. Impairments in emotional empathy have been mostly studied in people with neurological diseases with relatively diffuse injuries, such as traumatic brain injury, autism, and dementia [5]. Lesions of the amygdala caused by surgery or degenerative disease can impair emotional facial expression recognition, particularly for fearful expressions [6]. Neuroimaging studies in healthy participants have described a large increase in amygdala activity while individuals view photos of faces with fearful expressions [7, 8] and other emotionally valenced stimuli [9–12]. As individual studies have used varying paradigms and are limited by statistical power and sensitivity, it is still unclear whether the left or right amygdala is more consistently involved in emotional processing. Across studies, the left amygdala appears to be activated more often than the right amygdala, suggesting different roles for the left and right amygdalae in emotional processing [13]. Some studies have highlighted sex as a potential modulatory factor in emotional processing and its underlying neural mechanisms, and more broadly, the need to consider individual differences in understanding the neurobiology of emotion [14]. One study, which used quantitative meta-analysis, found no evidence to support amygdala lateralization as a function of sex or valence. Instead, their findings provided strong support for a functional dissociation between the left and right amygdala in terms of temporal dynamics [15].

Several fMRI studies have found increased amygdala activation in relation to self-reported anxiety and depression [16–18]. One common probe of human amygdala function is the identification of emotional facial expressions. Results from several functional neuroimaging studies have suggested that in healthy adults, the amygdala responds more strongly to fearful faces than to other expressions, such as neutral or happy faces [8, 11, 12, 19, 20]. This pattern of activity is generally lateralized, with a greater response in the left amygdala for standard face presentations. However, when facial expressions are masked so that the initial emotion is not consciously perceived, the right amygdala shows greater activation [8, 12, 20]. Additionally, adults with amygdala lesions exhibit deficits in their ability to recognize certain facial expressions, especially fearful expressions [6, 21–24]. In recent years, many researchers have investigated the role of the amygdala in emotional processing, learning, and memory. While most reports have emphasized the role of the amygdala in emotional processing, few have examined the differential contributions of the right and left amygdalae to affective information processing [25]. Many studies have observed lateralized amygdala activity, which is indicative of a hemisphere-specific processing difference between the left and right amygdalae. However, the direction of the reported lateralization is inconsistent [26]. In one study, the researchers hypothesized that in females with Turner syndrome, the right and left amygdalae play distinct but complementary roles that influence somatic and cognitive responses to emotional stimuli [27]. Right amygdala activation is associated with autonomic arousal, which indirectly influences cognitive processing centers in the left hemisphere, whereas left amygdala activation is linked to cognitive processing and recognition of emotional stimuli [27]. Some researchers have proposed that the left amygdala is more closely related to affective information encoding with a higher affinity for detailed feature extraction, while the right amygdala has a higher affinity for retrieval of pictorial or image-related affective information. Thus, the right amygdala may be involved in a faster but more superficial analysis of emotional stimuli compared with the left amygdala. These findings indicate that the left amygdala is more closely involved in language processing, while the right amygdala is more strongly engaged during fast, superficial, or gross analyses of affect-related information [26].

Several investigations have produced evidence that SSRIs act via the attenuation of activation in brain regions responsible for emotion processing. Such data provides support for the potential of fMRI with pharmacological probes as a way to identify the specific therapeutic effects of pharmaceutical agents in patients with anxiety and mood disorders [26]. For instance, one study showed that both escitalopram and citalopram decreased BOLD-signal activation in the amygdala and parahippocampal cortex in response to emotionally significant stimuli [26]. Another study indicated that antidepressant treatment reduces left limbic, subcortical, and neocortical capacity for activation in depressed participants and increases the dynamic range of the left prefrontal cortex. Changes in anterior cingulate function associated with symptomatic improvements indicate that fMRI may be a useful surrogate marker of antidepressant treatment response [28]. Fu et al. demonstrated that patients with depression had decreased neural responses to happy facial expressions in limbic, subcortical, and extrastriate cortical regions compared with healthy volunteers [29]. However, Arnone et al. showed that aberrant amygdala activation in response to sad facial emotions was specific to the depressed state, making it a potential biomarker for negative affective bias during a depressive episode [30]. Victor et al. suggested that antidepressant drugs exert their primary therapeutic mechanism by normalizing negative bias in information processing. This hypothesis was based partly on the finding that short-term administration of citalopram in healthy individuals enhanced the amygdala response to happy faces, while in depressed individuals a single-dose of reboxetine enhanced the behavioral responses to positively valenced stimuli [31].

In the present study, which had a longitudinal design, we used an emotion processing task during fMRI to examine whether abnormal amygdala responses to emotional stimuli were related to specific emotions or changes in the response of depressed participants to their antidepressant treatment. In addition, we investigated whether neural activity would differ over time between the right and left amygdalae in response to emotional stimuli. The aim of this study was to analyze the relative differences in neural activity between the right and left amygdalae in currently depressed patients during exposure to emotional stimuli in pre- and post-test (escitalopram) conditions. Most previous studies have investigated changes in neural responses in the amygdalae, either right or left, elicited by explicitly presented stimuli, or the differential amygdala response to facial stimuli. To the best of our knowledge, this is the first study to find a relative difference in neural activities between the left and right amygdala in individuals with depression. These findings could lead to useful parameters or biomarkers for depressive symptoms and treatment response.

## Methods

### Participants

The participant group comprised 20 individuals aged 28–55 years with a mean and standard deviation (SD) of 45.71 ± 8.04 who were not currently receiving medication for depression (N = 20). Previously, most of the participants had received antidepressants (escitalopram) for 6 weeks and several had been treated with hypnotics (Estazolam). The participants reported no co-morbid conditions. The participants were recruited from the outpatient clinic at the Department of Psychiatry, Chiayi and Wanqiao Branch, Taichung Veterans General Hospital, Chiayi, Taiwan. Participants underwent screening that included their medical and psychiatric history, laboratory testing, drug screening, a physical examination, and a neuromorphological MRI. Psychiatric diagnosis of depression was established using the Structured Clinical Interview from the DSM-IV and a semi-structured interview conducted by a research psychiatrist. Severity of illness was established using the 17-item Hamilton Depression Rating Scale (HDRS). One clinician assessed all participants. Patients with HDRS scores ≥18 were eligible for this study. Participants were excluded if they had (1) a moderate to severe suicidal risk; (2) major medical or neurological disorders, such as stroke, tumors, or Alzheimer’s disease; (3) substance abuse or dependence (including alcohol) within the past 6 months; (4) pregnancy, risk of pregnancy, or lactation; (5) general MRI incompatibility; (6) any history of a psychotic disorder, including bipolar I/II. After receiving a complete explanation of the study procedures, all participants provided written informed consent as approved by the institutional review board. This study was approved by the ethics committee of Taichung Veterans General Hospital and conducted in accordance with Good Clinical Practice procedures and the current revision of the Declaration of Helsinki [32].

### Antidepressant drug treatment

**Figure 1:**
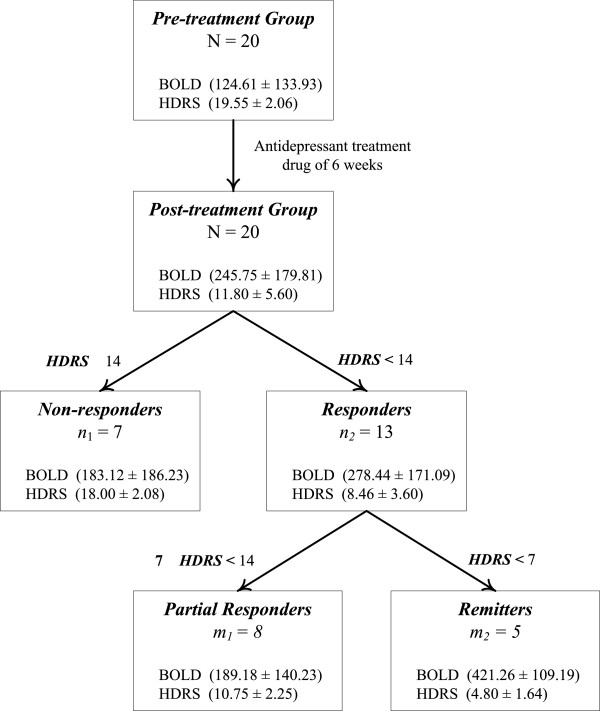
**Patient characteristics.** A total of 20 (N) patients were enrolled. Among them, a total of 20 participants (100%) completed an evaluation at week 6. Among them, a total of 13 (*n*_2_) participants (65%) had a response to the pharmaceutical treatment and five (*m*_2_) participants (25%) were in remission at week 6. The patient characteristics are summarized.

After the initial assessment, we conducted a baseline MRI scan and prescribed antidepressants (escitalopram) to each participant (visit 1). They returned for a second scan (visit 2) after 6 weeks. The participants were maintained on stable dosages (20 mg) throughout the 6-week period (Figure 1).

### Emotional processing task

**Figure 2:**
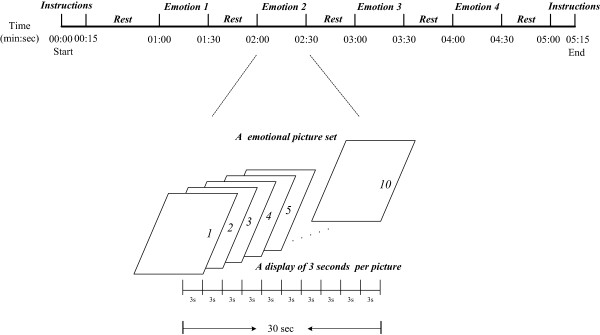
**Design of the emotion processing task.** Emotion *i* (*i* = 1,…,4) represents a certain emotional picture set comprising 10 pictures of happiness (H), sadness (S), fear (F), or disgust (D). Each picture was randomly displayed for 3 seconds, giving each emotional picture set a total presentation length of 30 seconds. The time intervals for each set of instructions and each rest were 15 and 30 seconds, respectively. The total task length was 5 minutes and 15 seconds.

Participants underwent fMRI while completing an emotion processing task (Figure 2). The task consisted of the blocked presentation of colored pictures from the International Affective Pictures System (IAPS). The IAPS images, including four emotional picture sets featuring images of happiness (H), sadness (S), fear (F) and disgust (D), were generated by a Pentium dual core PC and presented on a back projection screen that was visible to the participant via a mirror attached to the head coil. Each emotional picture set comprised 10 pictures and each picture was randomly displayed for 3 seconds, giving each emotional picture set a total length of 30 seconds. We presented stimuli in 11 time intervals, which included the instructions at the start and end of the task, four emotional picture sets, and five time intervals between the instructions and each emotional picture set. The total task length was 5 minutes and 15 seconds. The display of the video and the fMRI scanning was triggered at the same time for synchronous signal recording.

### fMRI data acquisition

Images were obtained using a 1.5-T Signa HDx system (GE Healthcare, United Kingdom) with a standard RF receiver head coil. A total of 105 fMRI images were acquired in each task period. To provide an anatomical framework for analysis of the fMRI images, we obtained high-resolution anatomical images using a gradient-echo EPI imaging sequence (TR = 3000 ms; TE = 50 ms; matrix: 64 × 64), which created transversal whole-brain acquisitions (26 slices, 4 mm slice thickness, 0 mm interslice gap). In addition, we conducted a coronal three-dimensional (3D) gradient-echo T1-weighted sequence (matrix: 256 × 256, TR = 12.4 ms, TE = 5.2 ms, 132 slices).

### Image calibration and amygdala tracing

Functional imaging analyses and amygdala tracings were performed using the general linear model with Statistical Parametric Mapping (SPM, Wellcome Trust Centre for Neuroimaging, London, England; http://www.fil.ion.ucl.ac.uk/spm). During fMRI scanning, unplanned head movements can result in inaccurate localization of activity. SPM enables every slice in an fMRI image sequence to be realigned for motion correction of individual head movements, and the resliced image sequence to be generated. Whole-brain fMRI volumes were realigned to the first volume, coregistered to each participant’s anatomical data, and normalized to fit a standard brain template (structural T1 images).

### 3D registration

**Figure 3:**
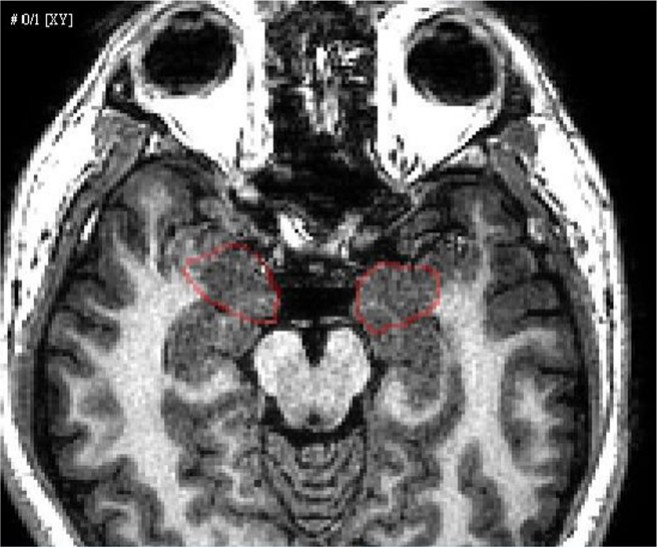
**Reconstruction of 3D models of the brain and skull.**

We used the coregistered MRI and fMRI image sequences to construct, spatially register, and fuse the 3D models of fMRI and MRI data using the Avizo system (VSG Inc., USA). Avizo is a type of commercial software that enables users to perform interactive visualization and computation on 3D data sets. We developed a procedure for amygdalae tracing and evaluating using the Tool Command Language (Tcl) in Avizo. A senior physician identified the amygdalae from the virtual MRI slices of the reconstructed 3D model (Figure 3). The spatially-related locations of the amygdalae in the time sequence of the virtual fMRI 3D model were automatically placed to trace the neural responses of amygdalae activation.

### Temporal evolution of the neural responses to the task

**Figure 4:**
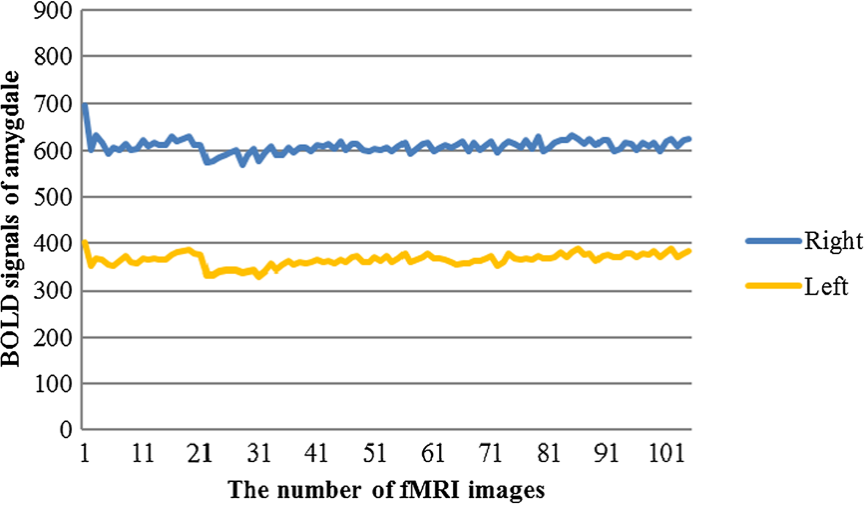
**Temporal evolution of the BOLD signal in the left and right amygdalae.** The time series was plotted against the magnitude of the BOLD signal. BOLD = blood oxygen level dependent.

The hemodynamic responses in the amygdalae during visual stimulation with emotional images varied in intensity. The magnitude of the blood-oxygen-level dependent (BOLD) signal was measured in each fMRI phase and the time course of the BOLD signal change plotted accordingly (Figure 4).

### Indexes for the evaluation of amygdalae activation

**Figure 5:**
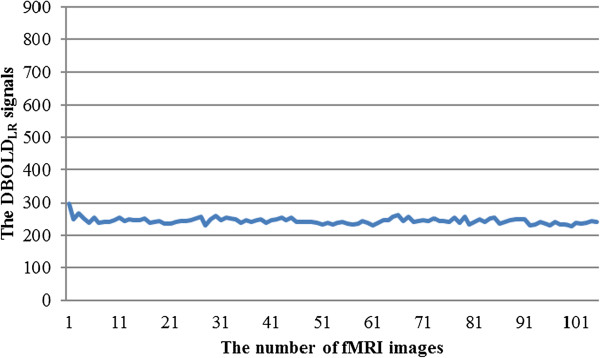
**Example of the DBOLD**_**LR**_ **signals for each fMRI image.** DBOLD_LR_: the difference in the BOLD signal between the right and left amygdalae.

We used the BOLD signal in each amygdala and the difference in the BOLD signal between the right and left amygdalae (*DBOLD*_*LR*_) to investigate amygdalae activation. The *DBOLD*_*LR*_ signal was computed by the following equation:


where *I*_*L*_ represents the BOLD signal in the left amygdala at a specific time point and *I*_*R*_ represents the BOLD signal in the right amygdala at a specific time point. The full time course of the *DBOLD*_*LR*_ signal is presented as a curve in Figure 5.

### Statistics

Our study had a two-factor design. Factor A had two levels: pre- and post-treatments, and Factor B had four levels: happiness, sadness, fear and disgust. We carried out a two-way ANOVA with repeated measures to 1) compare the BOLD signals and the *DBOLD*_*LR*_ signals in the pretreatment group with those in the post-treatment group, 2) test the BOLD signals and the *DBOLD*_*LR*_ signals among the four types of emotional stimuli, and 3) test the interaction between Factor A and Factor B using SPSS 17.0 statistical software. We used a paired *t* test to compare the HDRS scores in the pre-treatment group with those in the post-treatment group. We used a simple linear-regression method to analyze the relationship between HDRS scores and *DBOLD*_*LR*_ signals in the pre- and post-treatment groups.

## Results

**Figure 6:**
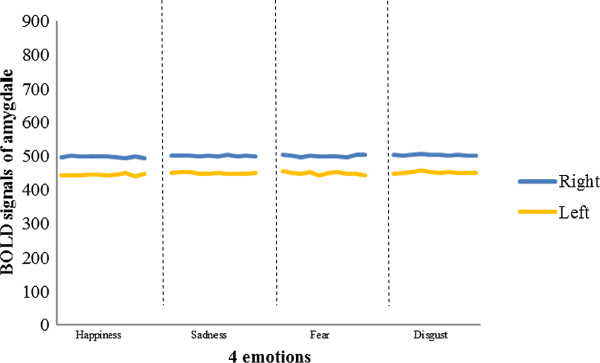
**Example of pre-treatment amygdala responses to four types of emotional stimuli in the left and right amygdalae.**

**Figure 7:**
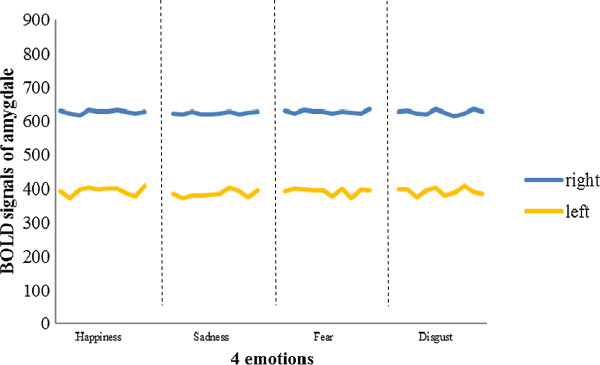
**Example of post-treatment amygdala responses to four types of emotional stimuli in left and right amygdalae.**

**Figure 8:**
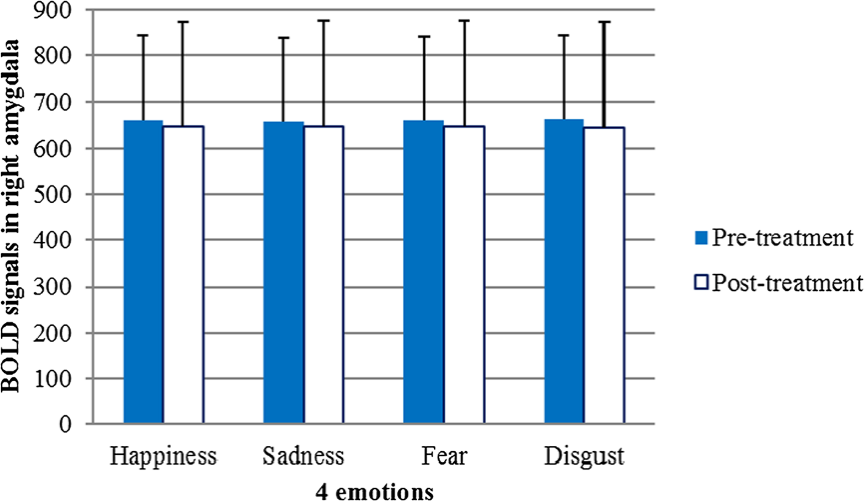
**Hemodynamic response to each of the four emotional stimuli after antidepressant treatment did not differ significantly from that before treatment in the right amygdala.** Error bars indicate standard deviations. BOLD = blood oxygen level dependent.

**Figure 9:**
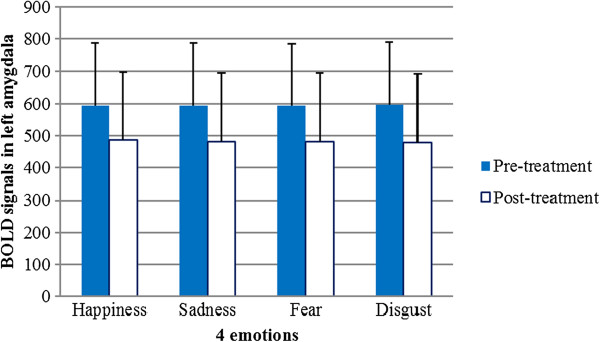
**Hemodynamic response to each of the four emotional stimuli after antidepressant treatment did not differ significantly from that before treatment in the left amygdala.** Error bars indicate standard deviations. BOLD = blood oxygen level dependent.

Figures 6 and 7 show the pre-treatment and post-treatment amygdala (right and left) responses to each of the four emotional stimuli, respectively. Among the 20 participants, six had greater pre-treatment responses in the left amygdala than in the right, and the remaining participants had greater pre-treatment responses in the right amygdala. Each individual showed lateralized amygdala activity, and the direction of asymmetry remained consistent throughout the treatment period. Seventeen participants exhibited an increase in the post-treatment BOLD signal, while the BOLD signal was attenuated in the other three. The results of a two-way ANOVA with repeated measures revealed that the BOLD signals in the amygdalae of the 20 participants were not significantly different between the pre- and post-treatment periods among the four types of emotional stimuli (Figures 8 and 9).

**Figure 10:**
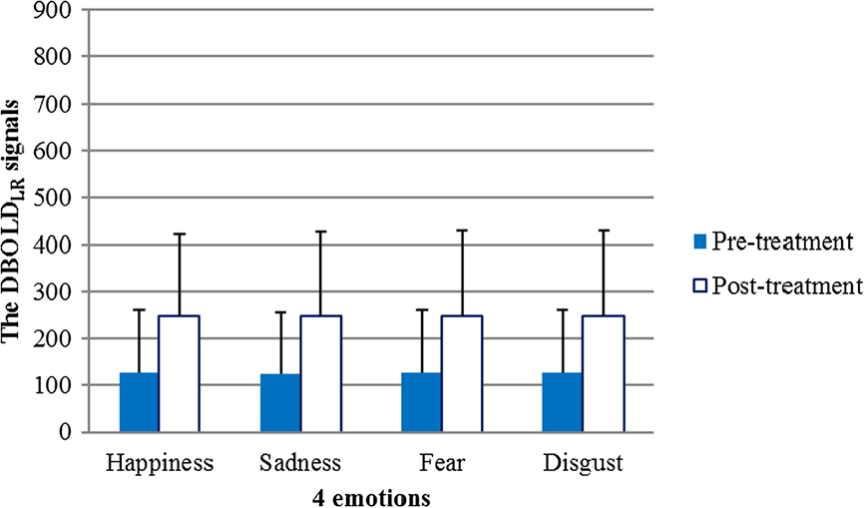
**DBOLD**_**LR**_ **signal responses to four emotional stimuli in the pre- and post-treatment groups.**

The means and standard deviations of the DBOLD_LR_ signals for all participants in the pre- and post- treatment groups are shown in Table 1. Compared with that in the pre-treatment group, the DBOLD_LR_ signals in the post-treatment group were greater in response to the four types of emotional stimuli (Figure 10). A two-way ANOVA with repeated measures revealed a significant effect of factor A on the DBOLD_LR_ signals (pre-treatment vs. post-treatment) (F = 9.350, *p* < 0.05), a non-significant effect of factor B on the DBOLD_LR_ signals (happiness, sadness, fear and disgust) (F = 0.608, *p* > 0.05), and no statistically significant interaction between factor A and factor B (F = 0.120, *p* > 0.05), as shown in Table 2.

**Table 1:** **The mean and standard deviation of DBOLD**_**LR**_ **signals response to four types of emotional stimuli in the pre- and post-treatment groups (N = 20)**

	***Happiness***	***Sadness***	***Fear***	***Disgust***
	***Mean ± SD***	***Mean ± SD***	***Mean ± SD***	***Mean ± SD***
*pre-treatment*	124.19 ± 134.50	122.76 ± 132.60	125.42 ± 134.53	126.05 ± 134.07
*post-treatment*	245.34 ± 175.25	245.22 ± 179.33s	245.86 ± 181.94	246.56 ± 182.73

**Table 2:** **Display of two-way ANOVA with repeated measures results (N = 20)**

Source of variation	Sum of squares	df	MS	F	***p*** -value	PES
*A* factors	586990.48	1	586990.48	9.350	0.006**	0.330
*B* factors	122.663	3	40.888	0.608	0.613	0.031
*A × B* interaction	26.275	3	8.758	0.120	0.948	0.006

df: degrees of freedom; MS: mean squares; PES = Partial Eta Squared; **: *p* < 0.05.

The DBOLD_LR_ signals of factor A revealed a significant difference between pre- and post-treatment groups. A factors represent pre- and post-treatment, and B factors represent four emotional stimuli. The *p*-value < 0.05 indicates a significant difference statistically.

The mean and standard deviation of HDRS score was 19.55 ± 2.06 in the pre-treatment group, and 11.80 ± 5.60 in the post-treatment group. A paired *t* test revealed significant differences in HDRS score between the pre- and post-treatment groups (*t* = 7, *p* < 0.05) (Figure 1). The HDRS scores of 13 participants after antidepressant treatment were below 14 with an average of 8.46 and a SD of 3.60. The mean and SD of the DBOLD_LR_ signals was 278.44 ± 171.09 (*n*_2_ = 13). The other seven patients had post-treatment HDRS scores that were greater than or equal to 14 (*n*_1_ = 7), an average HDRS score of 18.00 ± 2.08, and an averaged DBOLD_LR_ signal of 183.12 ± 186.33 (Figure 1). Thirteen of the 20 participants were placed into a separate group based on degree of response to antidepressants. The partial response group had an averaged HDRS score of 10.75 ± 2.25 and an averaged DBOLD_LR_ signal of 189.18 ± 140.23 (*m*_1_ = 8), and the remitted group had an averaged HDRS score of 4.80 ± 1.64 and an averaged DBOLD_LR_ signal of 421.26 ± 109.19 (*m*_2_ = 5). These data indicate that the greater the clinical response to antidepressant treatment, the greater the increase in neural activity in the amygdala.

**Figure 11:**
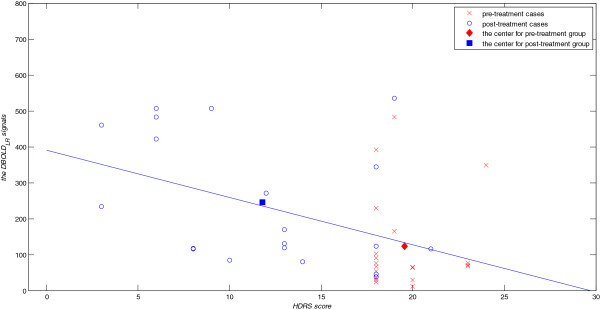
**Scatter plot of DBOLD**_**LR**_ **signals versus HDRS scores for the pre- and post-treatment groups.** The red cross (x) indicates the DBOLD_LR_ signals versus the HDRS scores obtained by the 20 participants in the pre-treatment group. The blue circle (o) indicates the DBOLD_LR_ signals versus the HDRS scores obtained by the 20 participants in the post-treatment group. The red diamond (◆) and blue square (■) represent the centroids of the pre- and post-treatment groups. The linear regression line is represented by y = -13.139x + 390.392 (R^2^ = 0.203, F = 9.662, *p* = 0.004). DBOLD_LR_: the difference in the BOLD signal between the right and left amygdalae.

Figure 11 shows a scatter plot of the DBOLD_LR_ signal versus the HDRS scores for the pre- and post-treatment groups. The red cross (x) marks the DBOLD_LR_ signals versus the HDRS scores of the 20 participants in the pre-treatment group. The blue circle (o) marks the DBOLD_LR_ signals versus the HDRS scores of the 20 participants in the post-treatment group. We conducted a linear regression analysis with the following linear regression equation: y = -13.139x + 390.392 (R^2^ = 0.203, F = 9.662, *p* = 0.004). The purpose was to assess the relationship between HDRS scores and the DBOLD_LR_ signal. The centroids of the pre- and post-treatment groups revealed that improved HDRS scores were correlated with increased DBOLD_LR_ signals, which implied an increase in the hemodynamic response difference between the right and left amygdala.

## Discussion

A computational neural network model of emotional information processing suggests that this process involves sustained amygdala activity elicited by the negative features of stimuli [33]. One study found that depression patients whose sustained reactivity to emotional stimuli was high in the amygdala exhibited the strongest improvements with cognitive behavioral therapy (CBT) [34]. Some studies have reported that depression patients exhibit hyperarousal in the left amygdala, even when processing stimuli outside conscious awareness. This increased amygdala activation tends to normalize with antidepressant treatment [35]. Gaffrey reported a strong positive relationship between major depressive disorder (MDD) severity and activity level in the right (*r* = .709, *p* = .007) rather than the left (*r* = .421, *p* = .099) amygdala during the presentation of sad faces [36]. In our study, we found that six depressive patients had greater activation in the right amygdala and 14 depressive patients had greater activation in the left amygdala prior to antidepressant treatment. In cases where patients had higher activation in one amygdala before the treatment, this persisted after the treatment. The mean values of BOLD signals in the left amygdala elicited by four emotional stimuli were clearly lower after the treatment (Figure 9). This decrease in BOLD signals implies an improvement in depressive symptoms resulting from the antidepressant treatment, according to some studies [35]. The large variance in BOLD signals among the 20 participants resulted in no statistically significant differences between the pre- and post-treatment groups. However, a bilateral difference in DBOLD_LR_ signals emerged when symptoms improved in response to medication. Our pre-treatment findings were inconsistent with those of a previous study by Gaffrey and colleagues [36]. This inconsistency may be explained by the following: 1) sample size and age differences: 20 adults participated in our study, whereas 11 preschool children were enrolled in Gaffrey’s study; 2) study aim: our study examined patterns of amygdala responses to emotional stimuli before and after antidepressant treatment; and 3) regression model: we analyzed the relationship between HDRS scores and the difference in response between the right and left amygdalae, whereas Gaffrey’s study analyzed the relationship between MDD severity and activity level in the right vs. left amygdala.

Peluso et al. demonstrated greater amygdala activation, in both the right and left amygdalae, in unipolar depressed individuals compared with non-depressed healthy individuals. In their study, increased depressive symptoms were associated with stronger amygdala activation [31]. Our findings may differ from those of Peluso et al. for the following reasons: 1) differences in sample size between our study and Peluso’s study; 2) more severe or persistent forms of mental illness may be related to pathological alterations of the amygdala structure; and 3) several patients reported partial remission (HDRS > 7) after treatment in our study, potentially contributing to the observed statistical bias.

Many researchers have investigated lateralization of amygdala activity during emotion processing tasks in people with various forms of mental illness. Most of these reports suggest that amygdala reactivity to negative facial stimuli is exaggerated in people with depression. Among these studies, Suslow and colleagues produced a representative report showing how depressed patients exhibit increased right amygdala reactivity in response to masked negative facial emotions and decreased activity in response to masked positive facial emotions, in contrast to healthy comparison participants [35]. Therefore, there appears to be a negative correlation between severity of depression and right amygdala reactivity in response to happy facial stimuli.

In this study, we investigated pre- and post-treatment responsiveness in the left and right amygdalae. Like previous studies, we found a normalization of amygdala responses after antidepressant treatment, but no statistically significant unilateral differences in BOLD signals in the pre- and post-treatment amygdalae. Various factors may have affected our findings, such as severity of illness at the time of inclusion in the study, level of response, previous treatment, previous number of episodes, and use of medication, e.g. benzodiazepines. Additionally, we only assessed changes within a population of depressed individuals, and did not have a control group. However, to the best of our knowledge, we are the first to report a negative correlation between disease severity and right-left differences in amygdala response. Thus, we suggest that unilateral differences in BOLD signal may be useful as an index or marker of depression. Future studies may reveal more about individual variations in response to emotion stimuli.

## Conclusions

We sought to examine changes in amygdala activity in response to antidepressant treatment. We found a significant difference in unilateral BOLD signals between our pre- and post-treatment period. Specifically, 16 participants exhibited enhanced unilateral differences in BOLD signals after antidepressant treatment, with decreased HDRS. However, four participants showed no significant differences in BOLD signals or HDRS after antidepressant treatment. Depression is a heterogeneous disorder with a highly variable course, an inconsistent response to treatment, and no established mechanism. This study presents one approach to understanding the biological mechanisms of major depression [35]. In the future, changes in BOLD signals as revealed by fMRI might be useful in evaluating the clinical manifestation of major depression.
